# Experimental Study on Corrosion and Mechanical Behavior of Main Cable Wires Considering the Effect of Strain

**DOI:** 10.3390/ma12050753

**Published:** 2019-03-05

**Authors:** Fangyuan Xu, Yuanli Chen, Xianglong Zheng, Rujin Ma, Hao Tian

**Affiliations:** 1 Zhejiang Institute of Communications, Hangzhou 311112, China; xfyalla@163.com (F.X.); yuanli@zjvtit.edu.cn (Y.C.); 2 Zhejiang Provincial Key Lab for Detection and Maintenance Technology of Road and Bridge, Hangzhou 311305, China; zxl_jky@163.com; 3 Department of Bridge Engineering, Tongji University, Shanghai 200092, China; rjma@tongji.edu.cn

**Keywords:** main cable wire, strain effect, super depth 3D microscopy technology, minimum sectional diameter, yield load, ultimate load

## Abstract

To study the corrosion degradation of cable wires in a bridge’s life, this research work created an accelerated corrosion test device, which sought to identify an optimal constant strain level. An accelerated corrosion test was carried out and the corroded specimens were scanned using super depth 3D microscopy technology. Mass loss and minimum cross-sectional diameter was measured to understand the degradation characteristics of cable wires at variable strains and corrosion time. The variation of elastic modulus, yield load, and ultimate load of corroded wires, subjected to a tensile test, were analyzed. The experimental results illustrate that the average mass loss ratio of the corroded cable wires increases nonlinearly as corrosion time increases. The higher the stress level, the more serious the corrosion level. The minimum cross-sectional diameter has good correlation with corrosion time and stress level. The elastic modulus of wires does not change significantly with the increase of corrosion time. Yield load and ultimate load decreases with the increase of strain level, and the rates of decline under different strains are nonlinear.

## 1. Introduction

High-strength steel wires play a pivotal role in the construction of modern large-span suspension bridges. However, over time, the wires in the main cable corrode and degrade, causing safety hazards. Addressing this corrosion has become a hot issue in the engineering field worldwide. 

Scholars across the globe have carried out various experimental researches on the issue—from atmospheric exposure tests in natural environments [1] to accelerated corrosion tests in artificially simulated scenarios [2,3,4]. The issues of concern include the effects of external temperature [5], relative humidity [6], pH value [7,8], ion concentration [9], and integrity of the corrosion resistant coating [10] on the corrosion of steel wires. Since the stress of the main cable in suspension bridges is generally between 500 MPa and 600 MPa, researchers have studied the effects of strain levels on corrosion rate. Test results by Barton [11] and Nakamura [12,13] show that tension has little effect on steel corrosion, while a test by Li [14] and Yang [15] show that strain levels can accelerate the corrosion rate of steel wire. Recently, Wu [16] conducted an experiment that confers steel wire a high level of accuracy in the amount of stress applied, and demonstrates that stress levels have a significant effect on stress corrosion cracking. A critical part of these experimental studies was to ensure that the high-strength steel wire maintained a high-level strain accurately for a prolonged period. Quantitative approaches used to assess the corrosion degree of high-strength steel wires include electrochemical methods (for measuring parameters such as natural corrosion potential [17] and polarization resistance [18]) and physical methods (for measuring physical quantities such as diameter and mass). New methods such as optical fiber grating technology [19], ultrasonic sensing technique [20], scanning electron microscope [21], X-ray computed tomography [22], and ultra-depth 3D microscopy technology provide novel ways and ideas for corrosion feature characterization.

In terms of the mechanical properties of corroded wires, a large number of tensile tests were conducted to obtain empirical conclusions about strength, elastic modulus, and elongation. Vehovar [23] applied tensile testing of corroded steel wire on an actual bridge to estimate the strength of the steel wire with the corrosion rate. A test by Liu [24] showed that strength degradation of the steel strand increases as the corrosion degree increases, and strength decreases by three quarters after two-and-a-half years of exposure. The experimental results of an artificial climate simulation test [25] and an electrochemical acceleration test [26] conducted show a nonlinear relationship between the ultimate strength and corrosion rate of steel strands. 

In this paper, an accelerated corrosion test of high-strength steel wires under a constant strain was carried out. Scanned by using ultra-depth 3D microscopy technology, the corrosion degradation law of high-strength steel wires in the main cable was studied with different strain levels and corrosion times. Static tensile tests were performed to explore the correlation between the corrosion degree and mechanical properties of cable wires.

## 2. Accelerated Corrosion Test of Cable Wires with Constant Strain

### 2.1. Material and Specimens

In order to be consistent with actual engineering practice, the high-strength steel wire used in the test was purchased from the main cable supplier of Xihoumen Bridge (Jiangsu Fasten Steel Cable Co.,Ltd, Jiangyin, China). Composition of the steel wire specimen was 0.78C, 0.22Si, 0.75Mn, P < 0.025, S < 0.025, Cr < 0.2, Cu < 0.2, and its nominal diameter was 5.25 mm.

Two parameters were considered in the test, namely, strain level and corrosion time. A total of 16 sets of high-strength steel wire corrosion tests were designed. Each set contained three test specimens. The designed conditions of these specimens are shown in Table 1. Since the strain of the main cable in the actual operation is roughly between 2000 με and 3000 με, the test considered four strain levels that could be in line with the actual strain condition of steel wires. Corrosion time was set from 1 to 4 days, a total of four time scales. 

### 2.2. Precise Strain-Holding Device

**Figure 1:**
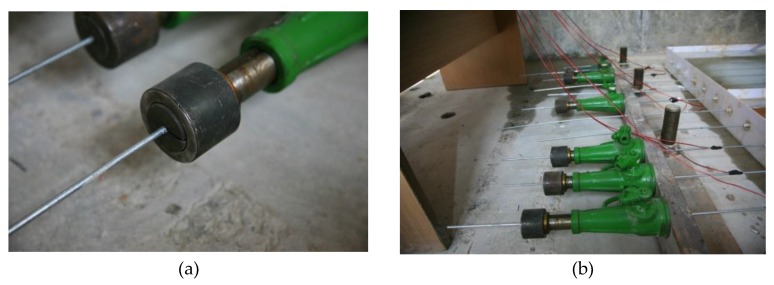
Strain-holding device based on wedge-type anchorage: (**a**) Specialized three-piece wedge anchorage for 5.25 mm wire; (**b**) perforated hydraulic jack to hold a strain.

Unlike the grip of ordinary steel bars, high-strength steel wire suffers from wedge sliding and insufficient gripping force owing to its small diameter, which makes it difficult to sustain constant strain for a prolonged period. In previous researches [9,27], a wedge-type anchorage was often used to maintain design stress. In the preliminary trial phase of our test, we adopted a three-piece wedge to hold a strain of high-strength steel wire, as shown in Figure 1. During the accelerated corrosion test, however, the galvanized anti-corrosion coating on the surface of the steel wire was found to have gradually become soft, thus causing a phenomenon of wedge sliding. The longitudinal displacement of the wedge-type anchorage resulted in quick attenuation of the high strain. Therefore, the preliminary trial using the wedge-type anchorage was abnegated.

**Figure 2:**
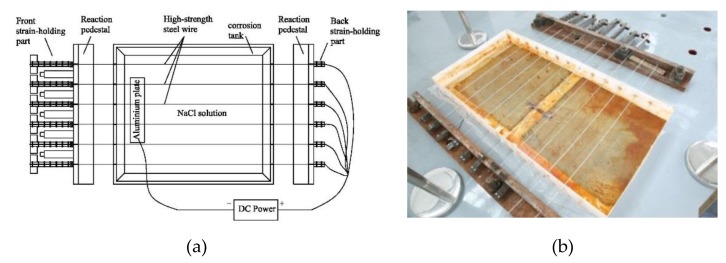
Diagram of the electrochemical accelerated corrosion device with high-tensile strain holding: (**a**) schematic diagram of the device; (**b**) actual experiment device.

**Figure 3:**
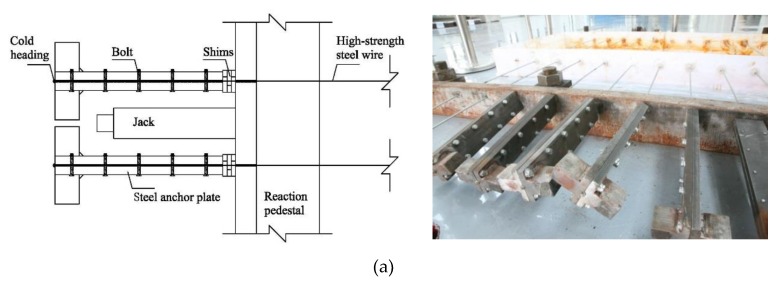

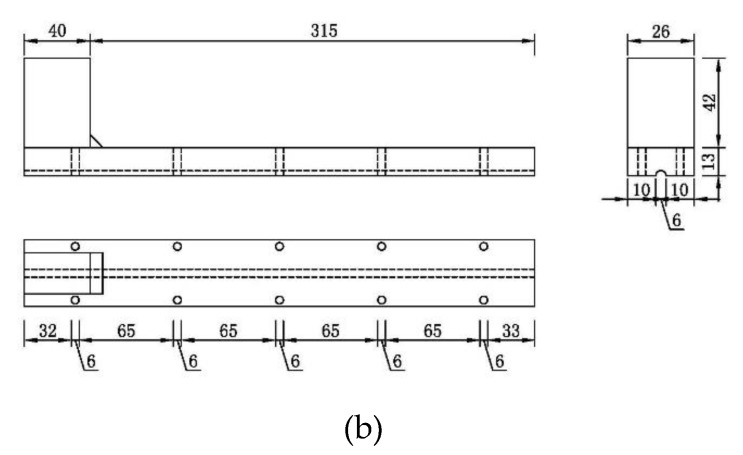
Front strain-holding part of the test device: (**a**) front strain-holding part; (**b**) anchorage plate (unit: mm).

**Figure 4:**
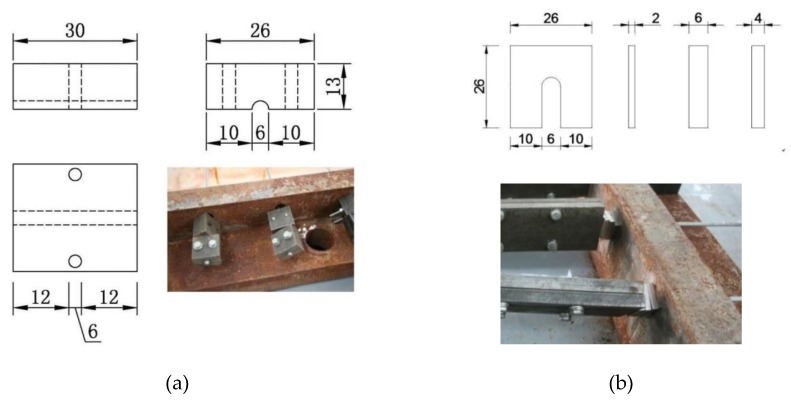
Back strain-holding part of the test device: (**a**) back strain-holding part (unit: mm); (**b**) shims (unit: mm).

To prevent the wedge from slipping, cold-drawn wires were fixed to the anchorage with button heads. At the same time, a constant strain to the steel wires was applied by hydraulic jack and steel shims. The accelerated corrosion device to test high-strength steel wires was divided into five components, namely, strain-holding part, corrosion tank, current loop, steel wire, and NaCl solution, as shown in Figure 2. In the top part of the strain-holding section, high-strength steel wires were stretched to the target strain by a jack, and a steel shim was inserted into its back. By jamming the button head, the target strain was expected to stay as is, and then the jack was removed. Figure 3 and Figure 4 show the front and back strain-holding sections, respectively. 

### 2.3. Electrochemical Accelerated Corrosion Test and Static Tensile Test

**Figure 5:**
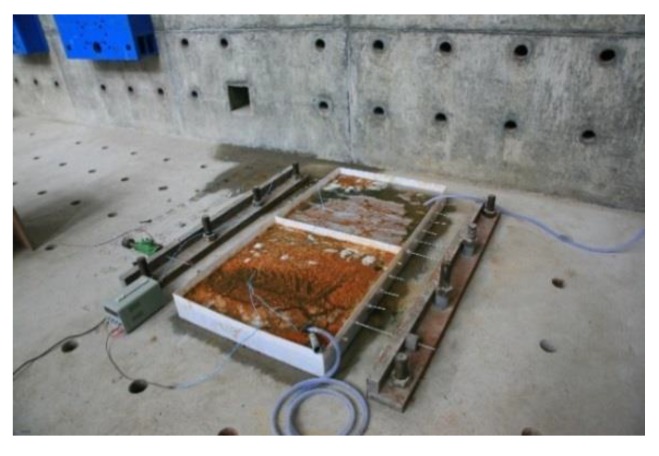
Electrochemical accelerated corrosion experiment.

High-strength steel wires were subject to different strains using the above-described strain-holding device that transfers the applied force to the ground anchor by a steel reaction pedestal. The corrosion tank was designed as a semi-closed solution tank containing a 5% NaCl electrolyte. To prevent sodium chloride solution from leaking and affecting the corrosion process, ABS plexiglass was selected as a substrate, and glass glue was reinforced in the gap. Figure 5 shows the current loop of the accelerated corrosion test. At the anode, all the steel wires passing through the reaction pedestal were connected in parallel in the circuit. At the cathode, the current was output to the two uniform aluminum plates. 

All specimens underwent an electrochemical accelerated corrosion test using the strain-holding device. After each batch of accelerated corrosion, in accordance with the “Standard for test method of long term performance and durability of ordinary concrete (GB/T 50082-2009)”, the wire specimens were pickled with a 12% hydrochloric acid solution, followed by rinsing in clean water. The specimens were then neutralized with Ca(OH)_2_ solution and rinsed again. After drying, they were stored in a desiccator for at least 4 h. A 50-cm corroded segment in the middle of the specimen was measured to identify the corrosion degree. 

**Figure 6:**
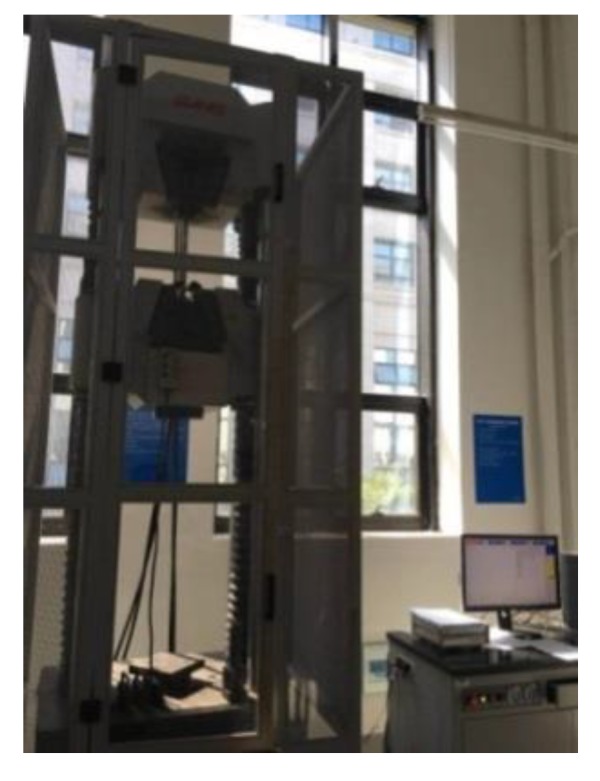
Static tensile testing of corroded wire.

The static tensile test was conducted on high-strength wire specimens using a servo-hydraulic testing machine (MTS Systems Corporation, Shanghai, China) and strain-control loading was applied, as shown in Figure 6. 

## 3. Analysis of Experimental Results

### 3.1. Morphology of Corroded Steel Wires

**Figure 7:**
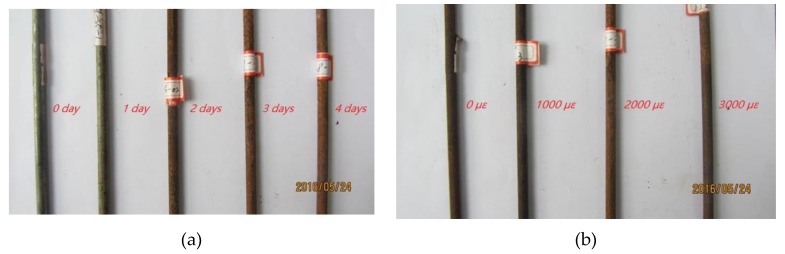
Corrosion shapes of cable wires: (**a**) Time-varying corrosion (at 2000 με strain); (**b**) strain-varying corrosion (on the third day).

Figure 7 shows the morphology of corroded steel wires under different corrosion times and strain levels. High-strength steel wires under strain of 2000 με gradually displayed zinc leaching from their galvanized coating, precipitation of dark-brown corrosion products, and enlargement of the corrosion area with increase in accelerated corrosion days. After accelerating the corrosion for three days, the corrosion products of steel wires under low strain levels were uniformly distributed in a longitudinal direction; however, products exposed to high strain levels displayed more severe corrosion and smaller nominal diameters.

**Figure 8:**
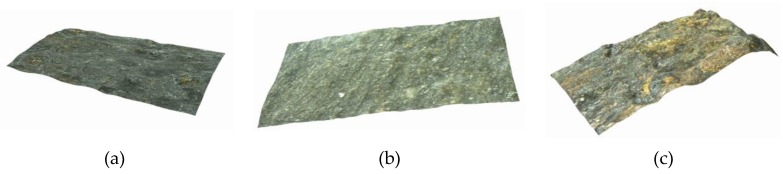
Comparison of the surface morphology after 1 day corrosion using ultra-depth 3D microscope with magnification of 500X: (**a**) 0 με; (**b**) 1000 με; (**c**) 2000 με.

In order to obtain more details of the morphology of corroded steel wire, high-strength steel wires were scanned using ultra-depth 3D microscopic imaging technology. The localized corrosion properties of high-strength steel wire with a macroscopic curvature cannot be accurately scanned in low magnification. Under high magnification, the depth from defocus (DFD) technology was used to deal with problems such as image edge shift and magnification fluctuation caused by focal length changes. The experiment found that magnifications of 500× and 1000× are decent enough to ensure observation of the morphology of the corroded surfaces of steel wire. A study under 500× magnification revealed that the surface morphology of the un-corroded steel wire was under 0 µε, and that of the corroded steel wires was 1000 με and 2000 με strains, respectively, after accelerated corrosion for one day (Figure 8). These results illustrate that the surface of the un-corroded steel wire had a well-preserved anti-corrosion coating and no exposed base material; several active corrosion pits are observed from the microscope image. In the case of the high-strength steel wire after accelerated corrosion under 1000 με strain for one day, the surface morphology was basically the same as that of the un-corroded wire, except that the number of active corrosion pits greatly increased and surface roughness was more prominent. For the high-strength steel wire after accelerated corrosion under 2000 με strain for one day, the corrosion products were obviously increased and the base material near the active corrosion pits showed outward precipitation, forming a contour of pitting corrosion.

**Figure 9:**
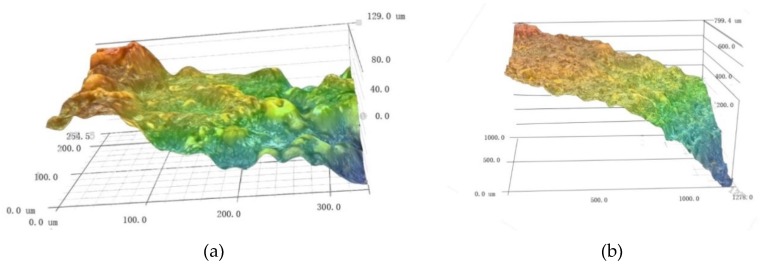
Comparison of the surface morphology under 2000 με strain: (**a**) accelerated corrosion for two days; (**b**) accelerated corrosion for four days.

Figure 9 shows the morphology of the high-strength steel wire with increasing corrosion time at 2000 με strain. It can be seen that the longer the corrosion time, the more severe the pitting on the surface of the steel wire was.

### 3.2. Mass Loss Ratio and Sectional Diameter of Corroded Steel Wire

**Figure 10:**
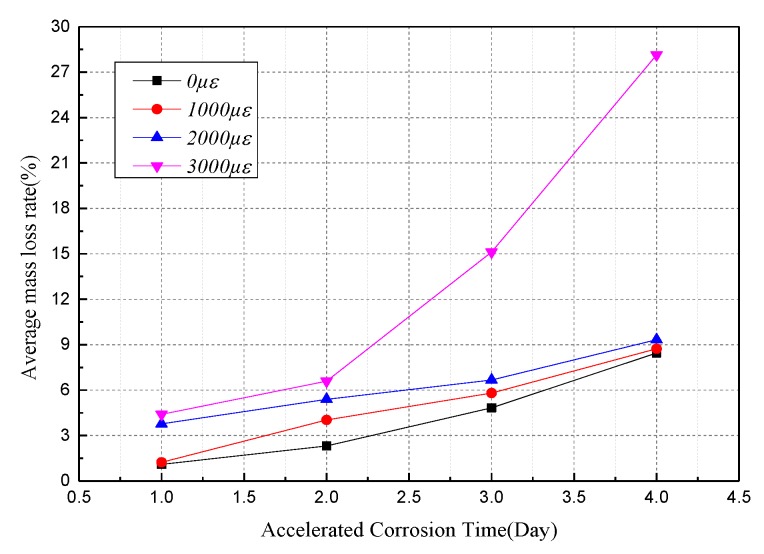
Average mass loss rates of steel wires under variable corrosion times and strains levels.

In order to quantitatively analyze the corrosion degree of the steel wires after accelerated corrosion, the corrosion of the high-strength steel wires was measured in terms of mass loss rate and minimum diameter of the section. By weighing specimens before and after the corrosion, the mass loss rate of the high-strength steel wires was obtained according to the following formula:

(1) $$\eta =\frac{m_{0}-m_{1}-m_{2}}{m_{0}-m_{1}}\times 100\%$$

where *m*_0_ is the wire weight before corrosion, *m*_1_ and *m*_2_ are the weights of the un-corroded segment and corroded segment of the rust-cleaning wire after the accelerated corrosion test, respectively. Table 2 shows the average mass loss rate of specimens under each specific strain and corrosion time. The tendency of the corrosion degree, as shown in Figure 10, shows that the average mass loss rate of steel wire increases nonlinearly with time and the corrosion rates have a faster-growing trend. It also illustrates that the higher the strain level, the more severe is the corrosion. On one hand, high-strength steel wires with high strain levels causes the substrate defects generated by pitting corrosion to stretch and expand continuously during the corrosion process, resulting in faster corrosion in a higher-strain steel wire. On the other hand, the high-strain state decreases the electrical resistance of the steel wire; when the external electrochemical potential is constant, it strengthens current density and intensifies the corrosion.

The corrosion of high-strength steel wire is normally not uniformly-distributed in a longitudinal direction. It may give rise to localized corrosion, the degree of which can be estimated indirectly by measuring the minimum diameter *d*_min_ with a vernier caliper. Thereby, the minimum diameter *d*_min_ of the wire section can be used as an index to measure the degree of pitting corrosion.

**Figure 11:**
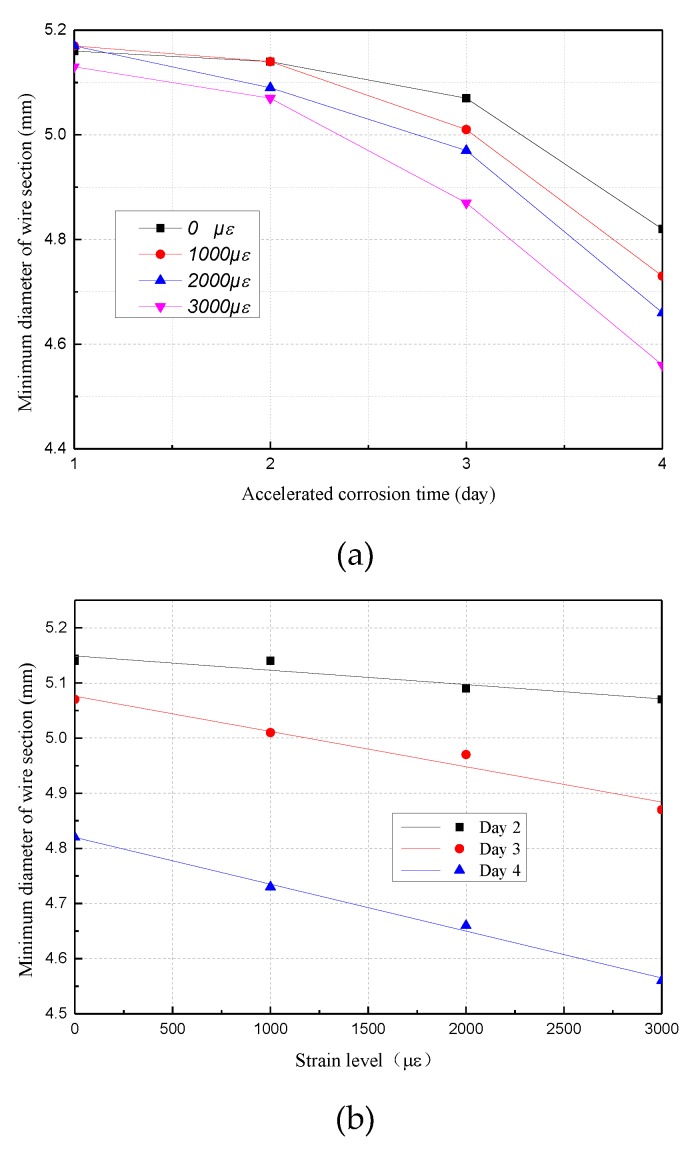
Curve of minimum diameter with corrosion time and strain level: (**a**) Minimum diameter over corrosion time; (**b**) minimum diameter over strain levels.

Figure 11a shows that the minimum diameter of the steel wires under different strain levels tends to decrease when the corrosion time increases. On the first couple of days, the curve tends to fluctuate, due to the influence of coating on the corrosion. Then, the reduction of the minimum diameter shows a non-linear growth rate. For specimens that were accelerated at a certain corrosion time, the minimum diameter of the steel wire was fitted to a linear function of strain, as shown in Figure 11b. This illustrates a good linear relationship between the minimum cross-sectional diameter and the applied strain, and the longer the time, the great the corrosion rate.

### 3.3. Mechanical Properties of Corroded Wires

**Figure 12:**
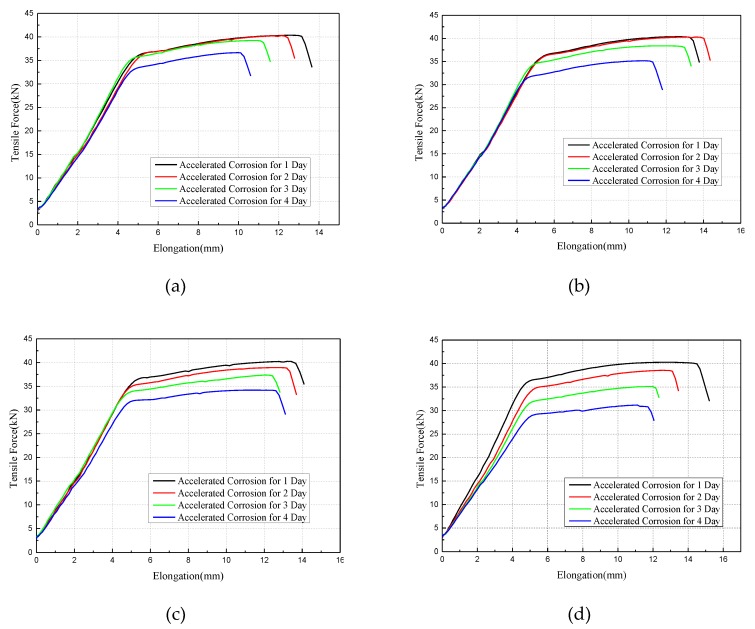
Force-elongation curve of corroded wires: (**a**) Under the strain of 0 με; (**b**) under the strain of 1000 με; (**c**) under the strain of 2000 με; (**d**) under the strain of 3000 με.

Figure 12 shows the results of the mechanical properties of the corroded wires subject to tensile tests. Based on the curve of tensile force and elongation, the yield loads of high-strength wires under small strain levels change slightly with the increasing corrosion time; however, it has an obvious variation of yield loads at 2000 με and 3000 με strain levels. The ultimate load and ultimate elongation of high-strength wires under the same strain level decrease with corrosion time, indicating a deterioration of the mechanical behavior of such wires due to the corrosion. According to Table 3, the yield load and ultimate load of steel wires present different levels of decline, when compared with the four applied strain levels. The result shows an obvious decline of cable wire strength under high strain levels.

The stress of the main cable wire in an actual suspension bridge is approximately 500 MPa to 600 MPa, which is about a strain of 3000 με. In Figure 12d, the slopes of the force—elongation curves corresponding to corrosion—for days 1 to 4 are 6.845, 5.891, 5.526, and 5.174, respectively, which when converted to the ratio of the elastic modulus is 100%, 91.56%, 94.97%, and 98.89%. This implies that the modulus of elasticity of corroded steel wires does not change significantly with increase of corrosion time.

## 4. Correlation Analysis between Corrosion Degree and Mechanical Properties of Cable Wires under Constant Strains

### 4.1. Tensile Behavior with the Average Mass Loss Rate

**Figure 13:**
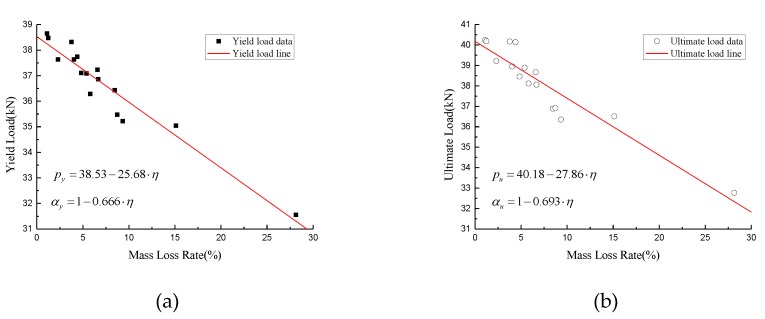
Mechanical properties of the specimens: (**a**) Yield load; (**b**) ultimate load.

According to Table 2 and Table 3, the mechanical properties of corroded steel wires are presented in Figure 13 with respect to mass loss rate. It can be seen that, as the average mass loss rate increases, the tensile behavior of the specimens decreases. By fitting the test data, the yield load and ultimate load can be expressed as:

(2) $$p_{y}=38.53-25.68\cdot \eta$$

(3) $$p_{u}=40.18-27.86\cdot \eta$$

where *p_y_* and *p_u_* are the yield load and ultimate load of high-strength steel wire, and η is mass loss rate of high-strength steel wires. The root mean squared error of the yield load is 0.5015, while the ultimate load produces an error of 0.7069. The ratio of yield load and ultimate load of corroded steel wires, applied to those of the non-corroded ones, can be obtained from Equations (2)–(3):

(4) $$\alpha_{y}=1-0.666\cdot \eta$$

(5) $$\alpha_{u}=1-0.693\cdot \eta$$

where α_y_ and α_u_ are the ratios for yield load and ultimate load. However, when compared with the test result in Reference [25,28,29], the slopes of the ratio, as shown in Table 4, are widely divergent, induced by the type of steel bars. 

### 4.2. Tensile Behavior with the Minimum Diameter of the Section

From Figure 10 and Figure 11, it can be seen that strain has a significant influence on the corrosion degree of high-strength cable wires, especially under the combined effect of uniform corrosion and localized pitting. Moreover, the change in the minimum diameter of the steel wire section can fairly describe the corrosion status. On the basis of polynomial regression analysis, the relationship between the minimum diameter and the corrosion influence factors is regressed, and the formula of the minimum cross-sectional diameter of steel wires under a specific strain is derived as follows:

(6) $$d_{\min}=\left(4.746+0.3845t-0.0915t^{2}\right)+\left(101-80.5t+8.5t^{2}\right)\cdot \varepsilon /10^{6}$$

where *d*_min_ is the minimum diameter of steel wire, *t* is the corrosion time in the test (unit: day) and ε is the constant stain applied to the steel wire (unit: με). 

**Figure 14:**
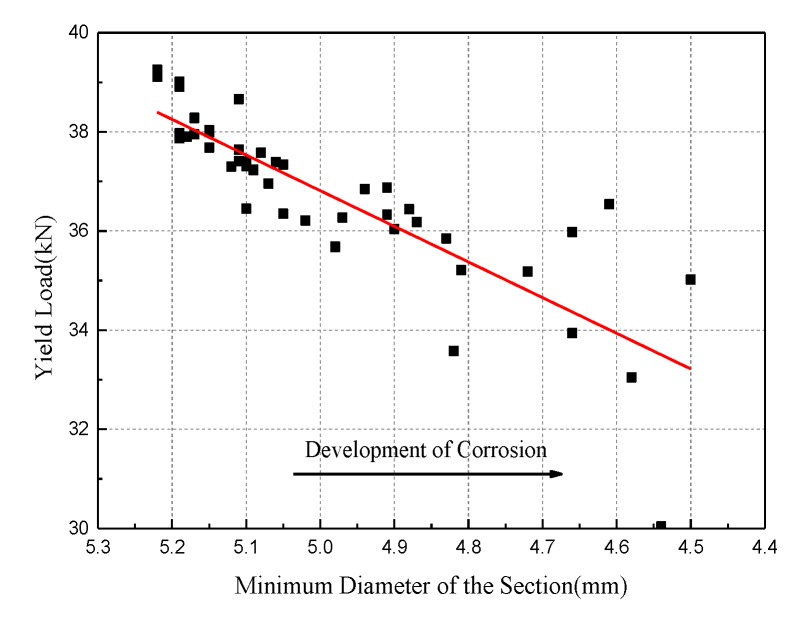
Variation of yield load versus minimum diameter.

Figure 14 shows the fitting result of specimens to the minimum diameter on the yield load of steel wire. The Pearson coefficient between the two parameters is 0.8427. In the initial stages of corrosion, the minimum diameter of the section is linear with the yield force. In the later stages of corrosion, however, the micro-cracks caused by pitting gives rise to discreteness of the data. The linear relationship between the minimum diameter and the yield load of steel wires can be expressed as:

(7) $$p_{y}=7.191d_{\min}+0.855$$

Then, by substituting the fitting expression (6), the variation of the yield force with the corrosion time can be obtained at a certain strain level:

(8) $$p_{y}=\left(34.98+2.77t-0.66t^{2}\right)+\left(7.26-5.79t+0.61t^{2}\right)\cdot \varepsilon /10^{4}$$

**Figure 15:**
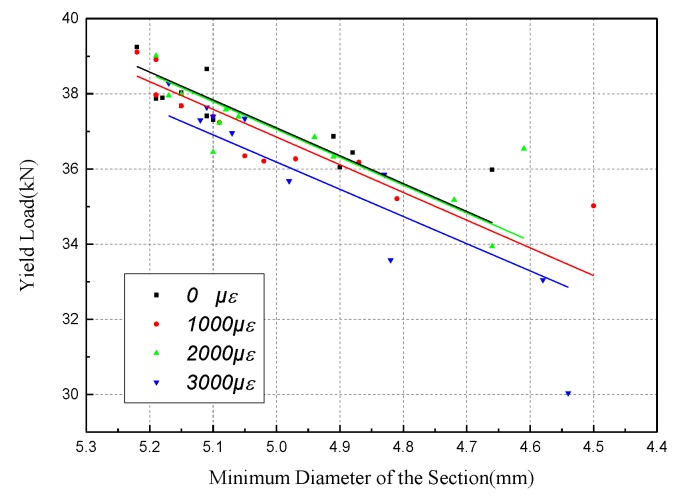
Correlation of minimum diameter and yield load for different strain levels.

The test data of the yield load is classified according to different strain levels, and the correlation of strain effect on the yield load is obtained, as shown in Figure 15. The slopes of the regression lines at the four strain levels are almost the same. In other words, the strain level only affects the minimum diameter of the section during the corrosion process, but has little effect on the decline rate of the yield load. 

It is worth noting that due to the concentration of corrosion in the wire section caused by high strain and localized pitting, the critical parameter related to the propagation of corrosion cracking is the minimum diameter of the section, rather than the mass loss rate, especially in the scenario of high strain and long-term corrosion. Therefore, it is unreliable to use the nominal diameter of the steel wire to calculate yield strength, and the data will be scattered as corrosion time increases.

**Figure 16:**
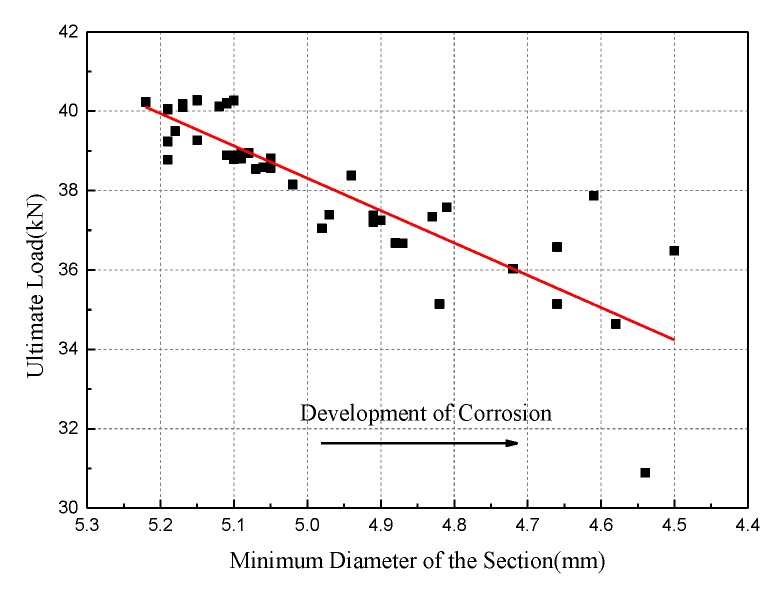
Variation of ultimate load versus minimum diameter.

Similar results can be found for the relationship between minimum diameter and ultimate load, as shown in Figure 16. The Pearson coefficient between the two parameters is 0.8605, and the regression formula is given as:

(9) $$p_{u}=8.148d_{\min}-2.429$$

Then, the variation of the ultimate force with corrosion time can be obtained at a certain strain level:

(10) $$p_{u}=\left(36.24+3.13t-0.75t^{2}\right)+\left(8.23-6.56t+0.693t^{2}\right)\cdot \varepsilon /10^{4}$$

**Figure 17:**
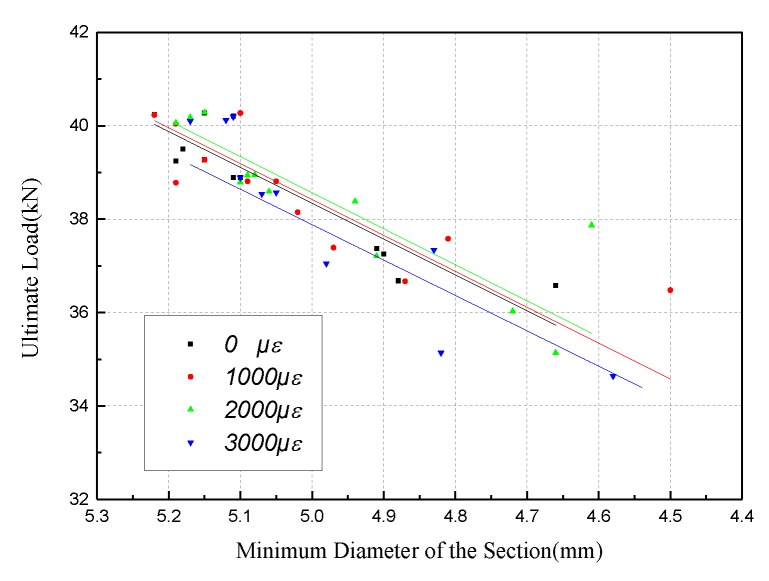
Correlation of minimum diameter and ultimate load for different strain levels.

Based on Figure 17, the slope of the ultimate force versus the minimum diameter remains the same as the strain level increases. That is to say, the strain level has little effect on the ultimate force with the deceleration rate of the minimum diameter.

## 5. Conclusions

This paper presents an experimental study on the corrosion of cable wires considering the effect of strain. The main conclusions are:

(1) A precise strain-holding device was designed, which changed the conventional way of force loading in the accelerated corrosion test. By jamming the button head, constant strain was applied to the steel wire by a hydraulic jack and steel shims. The corrosion of steel wires under different strain levels and corrosion times was obtained; the test result shows that the corroded product and corrosion morphology are different from ordinary steel bars. The longer the corrosion time, the larger the corrosion area. The diameter of the wire decreases at a non-linear rate as corrosion time increases. 

(2) A statistical analysis of the average mass loss rate and the minimum diameter of the wire section was carried out with respect to the strain level and corrosion time. This illustrates that the minimum diameter of the high-strength steel wire section has a linear correlation with the applied strain level, which can be used as a measure of corrosion degree.

(3) Based on linear fitting for tensile behavior, yield load and ultimate load can be expressed in terms of the mass loss rate of high-strength steel wires. However, when compared with the references, the slopes of the ratio of yield load and ultimate load of corroded steel wires to those of the non-corroded ones are widely divergent.

(4) A statistical regression analysis was carried out to obtain the expression of minimum diameter with various strain levels and corrosion time. Then, variation of the yield load and ultimate load was given with corrosion time and strain level. The result shows that stress level affects the minimum diameter of the section during the corrosion process, which indirectly reduces the yield load. 

## Figures and Tables

**Table 1:** Composition of specimens.

Specimens Items		Corrosion Time/Day			
		1	2	3	4
Strain level	0 με	0-1-1(/2/3) ^1^	0-2-1(/2/3)	0-3-1(/2/3)	0-4-1(/2/3)
	1000 με	1000-1-1(/2/3)	1000-2-1(/2/3)	1000-3-1(/2/3)	1000-4-1(/2/3)
	2000 με	2000-1-1(/2/3)	2000-2-1(/2/3)	2000-3-1(/2/3)	2000-4-1(/2/3)
	3000 με	3000-1-1(/2/3)	3000-2-1(/2/3)	3000-3-1(/2/3)	3000-4-1(/2/3)

^1^ The specimen number is in the form of “strain value—corrosion time—number of parallel tests”.

**Table 2:** Mass loss rate of corroded cable wires.

Strain Level	1 Day	2 Day	3 Day	4 Day
0 με	1.10%	2.31%	4.83%	8.45%
1000 με	1.23%	4.03%	5.81%	8.73%
2000 με	3.77%	5.39%	6.67%	9.33%
3000 με	4.40%	6.59%	15.12%	28.14%

**Table 3:** Mechanical property of corroded cable wires.

Mechanical Property	Strain Level	1 Day	2 Day	3 Day	4 Day
Yield Load (kN)	0 με	38.65	37.63	37.11	36.43
	1000 με	38.47	37.63	36.28	35.47
	2000 με	38.32	37.09	36.86	35.22
	3000 με	37.74	37.23	35.04	31.55
Ultimate Load (kN)	0 με	40.24	39.22	38.46	36.88
	1000 με	40.18	38.95	38.12	36.91
	2000 με	40.17	38.89	38.06	36.35
	3000 με	40.14	38.67	36.51	32.77

**Table 4:** Comparison of the ratio of yield load and ultimate load.

Researcher	Bar Type	Diameter/mm	Yield Load	Ultimate Load
Authors	Steel Wire	5.25	$\alpha_{y}=1-0.666\cdot \eta$	$\alpha_{u}=1-0.693\cdot \eta$
Wu et al. [28]	Steel Bar	16	$\alpha_{y}=1-1.625\cdot \eta$	$\alpha_{u}=1-1.775\cdot \eta$
Zhang et al. [29]	Bar in Concrete	6.5	$\alpha_{y}=1-12\cdot \eta$	$\alpha_{u}=1-1.36\cdot \eta$
Li et al. [25]	Steel Strand	15.20	-	$\alpha_{u}=1-2.683\cdot \eta$
